# *TACC3* overexpression in cholangiocarcinoma correlates with poor prognosis and is a potential anti-cancer molecular drug target for HDAC inhibitors

**DOI:** 10.18632/oncotarget.12254

**Published:** 2016-09-26

**Authors:** Jun-chuang He, Wei Yao, Jian-ming Wang, Peter Schemmer, Yan Yang, Yan Liu, Ya-wei Qian, Wei-peng Qi, Jian Zhang, Qi Shen, Tao Yang

**Affiliations:** ^1^ Department of Biliary and Pancreatic Surgery/Cancer Research Center Affiliated Tongji Hospital, Tongji Medical College, Huazhong University of Science and Technology, Wuhan, Hubei 430030, China; ^2^ Department of General and Transplant Surgery, University Hospital Heidelberg, Heidelberg 69120, Germany

**Keywords:** histone deacetylase (HDAC), HDAC inhibitors (HDACIs), microarray, transforming acidic coiled-coil-containing protein 3 (TACC3), cholangiocarcinoma (CCA)

## Abstract

Histone deacetylases (HDACs) have been implicated in multiple malignant tumors, and HDAC inhibitors (HDACIs) exert anti-cancer effects. However, the expression of HDACs and the anti-tumor mechanism of HDACIs in cholangiocarcinoma (CCA) have not yet been elucidated. In this study, we found that expression of HDACs 2, 3, and 8 were up-regulated in CCA tissues and those patients with high expression of HDAC2 and/or HDAC3 had a worse prognosis. In CCA cells, two HDACIs, trichostatin (TSA) and vorinostat (SAHA), suppressed proliferation and induced apoptosis and G2/M cycle arrest. Microarray analysis revealed that *TACC3* mRNA was down-regulated in CCA cells treated with TSA. *TACC3* was highly expressed in CCA tissues and predicted a poor prognosis in CCA patients. *TACC3* knockdown induced G2/M cycle arrest and suppressed the invasion, metastasis, and proliferation of CCA cells, both *in vitro* and *in vivo*. *TACC3* overexpression reversed the effects of its knockdown. These findings suggest *TACC3* may be a useful prognostic biomarker for CCA and is a potential therapeutic target for HDACIs.

## INTRODUCTION

Cholangiocarcinoma (CCA) originates from the epithelial cells of the intra- and extra-hepatic biliary trees and is the second most common hepatobiliary malignancy after hepatocellular carcinoma [1]. Morbidity due to CCA has been rising worldwide over the past several decades [2]. Due to the strong and early invasive characteristics of CCA, most patients are diagnosed in the later stages of disease. In addition, more than two-thirds of patients are ineligible for surgery, the only potential curative option. Unfortunately, even radical resection is associated with a high rate of recurrence that results in a five-year survival rate of less than 20-40%. Most CCA patients resort to palliative treatments, such as chemotherapy and radiotherapy, with a response of only 10-20% [3–5]. Therefore, it is crucial to find new therapeutic strategies and an effective prognostic biomarker for patients with CCA.

Tumor development requires both genetic and epigenetic alterations, which include DNA methylation, histone modifications, and gene silencing by small RNAs [6]. Acetylation of the N-terminal tail of core histones is tightly controlled by the antagonistic actions of histone acetyltransferases (HATs) and histone deacetylases (HDACs) [7]. HDACs remove acetyl groups from the lysine residues of histones, increasing the ionic interactions between DNA and histones, and resulting in chromatin condensation that represses transcription [8, 9]. As a consequence, excessive deacetylation of histones induces cell proliferation, angiogenesis, cell migration, and invasion by inactivating tumor suppressor genes [10]. There are eighteen HDAC isoforms categorized into four classes. Class I HDACs (HDACs 1, 2, 3 and 8) are the best characterized and are related to the yeast RPD3 deacetylase. Class II HDACs are subdivided into class IIa (HDACs 4, 5, 7 and 9) and class IIb HDACs (HDACs 6 and 10), which are homologous to the yeast Hda1 deacetylase. Class III HDACs (sirtuins [SIRTs]) include the seven subtypes of SIRTs (SIRTs 1-7), while class IV HDACs only includes HDAC11 [11–13]. Many studies have reported on the differential expression of class I and II HDAC isoforms in a variety of malignancies and cell lines, such as lymphoma [14], breast [15], gastric [16], colorectal [17], and lung cancers [18], hepatocellular carcinoma [19], as well as cholangiocarcinoma [20]. Despite the fact that class I and II HDACs are overexpressed in a variety of CCA cell lines, few studies have examined HDAC expression status and its prognostic value in CCA patients [20].

A multitude of natural and synthetic compounds function as HDAC inhibitors (HDACIs) that weaken histone-DNA interactions permitting a more open chromatin conformation and enhancing gene transcription. HDACIs as cancer treatments lead to increased transcription of tumor suppressor genes, the induction of cell cycle arrest, differentiation, and apoptosis, and the inhibition of angiogenesis [21, 22]. HDACIs have been classified into four groups according to their chemistry: hydroxamates (trichostatin [TSA] and vorinostat [SAHA]), cyclic peptides (romidepsin), aliphatic acids, and benzamides [21, 23]. Out of all of the HDACIs, SAHA has shown the most promising antitumor effects on tumor types at doses that are well-tolerated by patients and was the first HDACI approved by the U.S. Food and Drug Administration (FDA) for the treatment of cutaneous T-cell lymphoma [24]. TSA is a pan-HDACI and was the first natural hydroxamate found to inhibit cell proliferation and induce apoptosis in many cancer cell lines, such as breast cancer, lung cancer, and CCA cells [20, 21, 25]. Despite the broad application of HDACIs in cell culture, animal models, and early phase clinical trials, surprisingly little is known about specific anti-tumor mechanism of HDACIs in CCA.

Transforming acidic coiled-coil-containing protein 3 (TACC3), a member of the TACC family, is encoded by the *TACC3* gene, which is located on 4p16.3. TACC3 is a centrosome/microtubule-associated protein characterized by a highly conserved C-terminal coiled-coil domain [26, 27]. TACC3 regulates centrosome integrity and microtubule dynamics during mitosis, and has recently been shown to modulate epithelial-mesenchymal transition (EMT) through the activation of the PI3K/Akt and ERK signaling pathways in cervical cancer cells [28, 29]. TACC3 is also involved in the development of glioblastoma [30], multiple myeloma [31], lung cancer [32] and breast cancer [33], while *TACC3* expression is decreased in thyroid and ovarian cancers [34, 35]. The function of TACC3 and its relationship with HDACIs in CCA is unknown.

In the present study, we first investigated the expression of class I and II HDACs in CCA tissues, and then, assessed the correlation of HDAC expression with CCA patient clinicopathological characteristics. We then demonstrated that TSA and SAHA inhibited cell proliferation and induced apoptosis and cell cycle arrest in CCA cell lines. In addition, through a microarray experiment, we found that *TACC3* expression was down-regulated when cells were treated with HDACIs. Expression of *TACC3* and its correlation with the clinicopathological features of CCA were also investigated. Moreover, the functions of TACC3 were assessed by RNA knockdown and rescue experiments, *in vitro* and *in vivo*. Our findings suggest that HDAC2, HDAC3, and *TACC3* are highly expressed in CCA tissues and that their expression correlates with poor prognosis in CCA patients. Thus, *TACC3* may be a target of HDACIs, which inhibit the proliferation and migration of CCA cells.

## RESULTS

### High expression of HDAC2 and HDAC3 promotes tumor progression and correlates with poor prognosis

**Figure 1:**
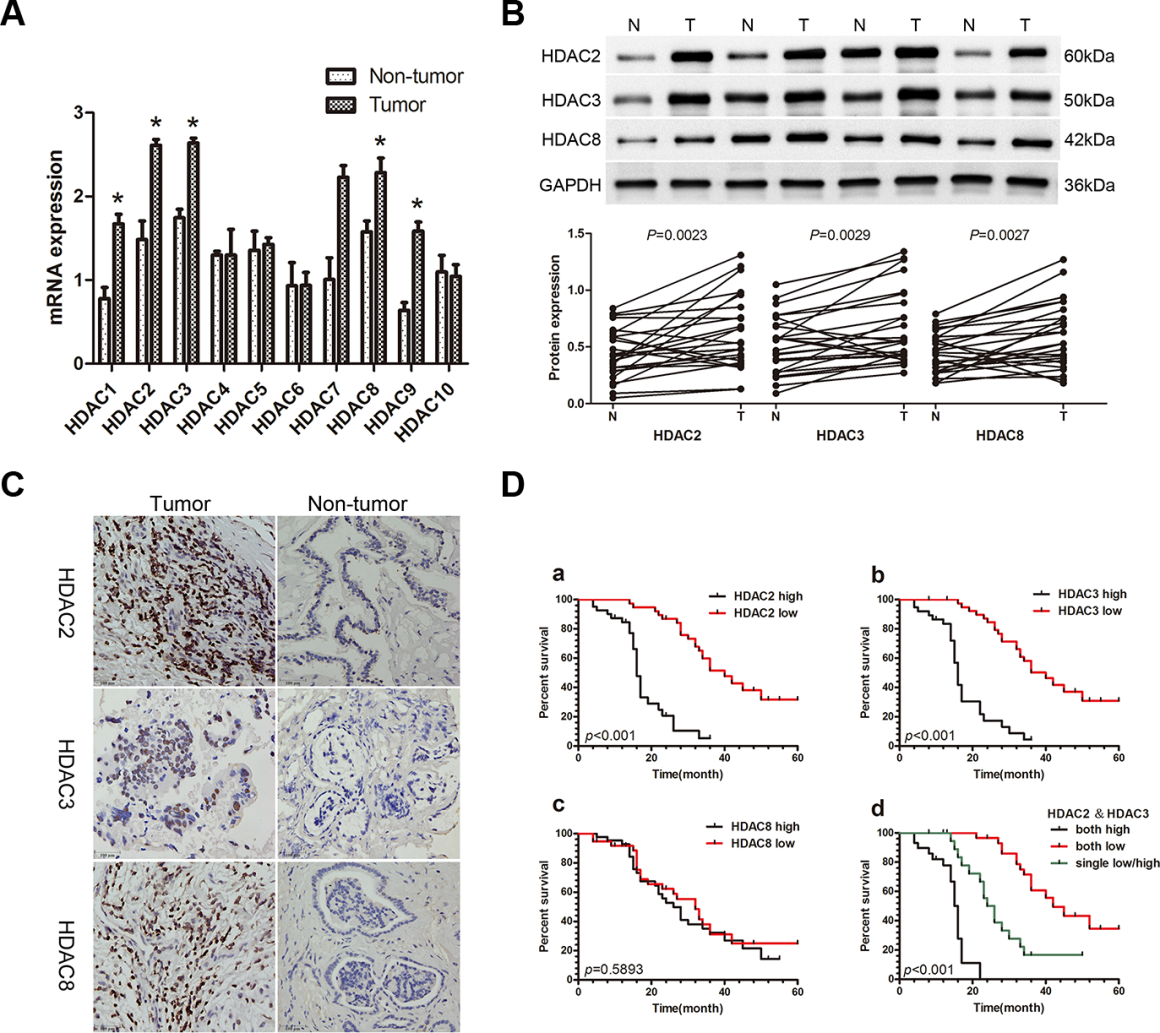
Expression of HDACs in patients with CCA and the correlation of the HDAC2/3 expression with poor prognosis **A.** qRT-PCR was used to measure the expression of class I and class II HDACs in 26 paired CCA and non-tumor tissue samples. Total RNA was isolated from at least three samples of each tissue and *ACTB* was used as the internal control. Fold changes were calculated through relative quantification (2^−ΔΔCt^). Data are shown as mean ± SD, *P<0.05. **B.** Western blot was used to detect protein expression of HDACs 2, 3, and 8. GAPDH was used as the internal control and all experiments were repeated three times. A representative image is shown (upper panel), and the statistical analysis of the relative optical density of each band is shown (lower panel). *P<0.05. **C.** Representative IHC staining of HDACs 2, 3 and 8 in CCA tissues and paired adjacent non-tumor tissue (400X). Scale bar, 100μm. **D.** Kaplan-Meier analysis. a. Patients with low HDAC2 expression (n=37) had longer overall survival (OS) than patients with high HDAC2 expression (n=42; median OS: 40 months *vs* 16 months, *P*<0.001, log-rank test). b. Patients with low HDAC3 expression (n=35) had longer OS than patients with high HDAC3 expression (n=44; median OS: 43 months *vs* 17 months, *P*<0.001, log-rank test). c. There were no differences in OS between the low HDAC8 expression group (n=37) and the high HDAC8 expression group (n=42; median OS: 32 months *vs* 26 months, *P*=0.5893, log-rank test). d. Patients with lower expression of both HDAC2 and HDAC3 (n=21) had a longer OS than patients with higher expression of both HDAC2 and HDAC3 (n=22), or low expression of either HDAC2 or HDAC3 (n=36; median OS: 42 months *vs* 16 months *vs* 25 months, *P*<0.001, log-rank test).

The expression of class I and class II HDAC mRNAs was assayed with qRT-PCR in 26 paired CCA and adjacent non-tumor fresh tissue samples. Among HDACs 1-10, class I HDACs (*HDAC1*, *HDAC2*, *HDAC3*, *and HDAC8*) and *HDAC9* were more highly expressed in CCA tissues compared with paired non-tumor tissues (*P*<0.05; Figure 1A). HDAC protein expression was then assayed by Western blot (WB), and the expression of HDAC2, HDAC3, and HDAC8 were higher in CCA tissues compared with paired non-tumor tissues (*P*<0.05; Figure 1B). We selected HDAC2, HDAC3, and HDAC8 to further examine in 79 paraformaldehyde-fixed, paraffin-embedded paired CCA and adjacent non-tumor tissues with immunohistochemistry (IHC). Expression of all three HDACs was localized to the nucleus (Figure 1C), and high expression of HDAC2, HDAC3 and HDAC8 was observed in 42 (53.16%), 44 (55.69%), and 42 (53.16%) CCA cases, respectively. CCA tissues had higher expression compared with corresponding matched non-tumor tissues (27.85%, 30.38%, and 32.91% for HDAC2, HDAC3 and HDAC8, respectively; χ^2^ =10.505, 10.327, 6.609; *P*<0.05; Table 1). Furthermore, high expression of HDAC2 and HDAC3 was positively correlated with lymph node metastasis (χ^2^ =15.474; 9.757) (*P*<0.001, *P*=0.002), TNM stage (χ^2^ =13.021; 7.693) (*P*<0.001, *P*=0.006), and differentiation (χ^2^ =9.125; 8.313) (*P*=0.003; *P*=0.004) in CCA; however, no such correlations were observed for HDAC8 (*P*>0.05; Table 1). Kaplan-Meier analysis showed that patients with low expression of HDAC2 and/or HDAC3 exhibited a longer overall survival (OS) than those with high expression of HDAC2 and/or HDAC3 (*P*<0.001, Figure 1D), but no correlation with OS was observed for HDAC8. Moreover, multivariate COX regression analyses identified HDAC2 and HDAC3 as significant, independent prognostic factors for OS (95%CI: 1.393-9.857, *P*=0.009; 95%CI: 2.419-12.575, *P*<0.001; Table 2), as well as for lymph node status (95%CI: 1.078-6.320, *P*=0.034), TNM stage (95%CI: 2.335-10.182, *P*=0.002), and differentiation (95%CI: 1.950-8.758, *P*<0.001; Table 2).

**Table 1:** Expression of HDAC2, HDAC3, HDAC8, and TACC3 in 79 CCA patients

Group	N	HDAC2		*P*	HDAC3		*P*	HDAC8		*P*	TACC3		*P*
		Low	High		Low	High		Low	High		Low	High	
**Age**													
** ≤ 60**	45	27	18	0.329	25	20	0.396	17	28	0.052	22	23	0.259
** > 60**	34	19	15		17	17		20	14		20	14	
**Gender**													
** male**	45	25	20	0.476	27	18	0.345	19	26	0.165	23	22	0.238
** female**	34	20	14		18	16		19	15		21	13	
**CCA/non-tumor tissues**													
** CCA**	79	37	42	0.001	35	44	0.001	37	42	0.01	34	45	0.008
** non-tumor**	79	57	22		55	24		53	26		50	29	
**Lymphoid nodal status**													
** No**	48	31	17	<0.001	28	20	0.002	25	23	0.245	27	21	0.003
** Yes**	31	6	25		7	24		12	19		7	24	
**TNM staging**													
** I-II**	45	29	16	<0.001	26	19	0.006	25	20	0.074	27	18	<0.001
** III-IV**	34	8	26		9	25		12	22		7	27	
**Differentiation**													
** Well**	27	19	8	0.003	18	9	0.004	16	11	0.111	19	8	<0.001
** Medium/Poorly**	52	18	34		17	35		21	31		15	37	

*p* values were calculated by Pearson's Chi-square test.

**Table 2:** Univariate and multivariate analyses for predictors of overall survival (OS)

Variables	OS		
	Univariate analysis	Multivariate analysis	
	*p* value	95% CI	*p* value
Age (>60years vs ≤60years)	0.141		
Gender (male vs female)	0.234		
Lymphoid nodal status (no vs yes)	<0.001	1.078-6.320	0.034
TNM stage (I-II vs III-IV)	<0.001	2.335-10.182	0.002
Differentiation (well vs medium/poorly)	<0.001	1.950-8.758	<0.001
HDAC2 expression (low vs high)	<0.001	1.393-9.857	0.009
HDAC3 expression (low vs high)	<0.001	2.419-12.575	<0.001
HDAC8 expression (low vs high)	0.5893		
TACC3 expression (low vs high)	0.0008	1.503–3.1670	0.0063

Abbreviations: CI, confidence interval.

### TSA and SAHA suppress cell proliferation, promote cell apoptosis, induce cell cycle arrest, and restrain EMT in CCA cell lines

**Figure 2:**
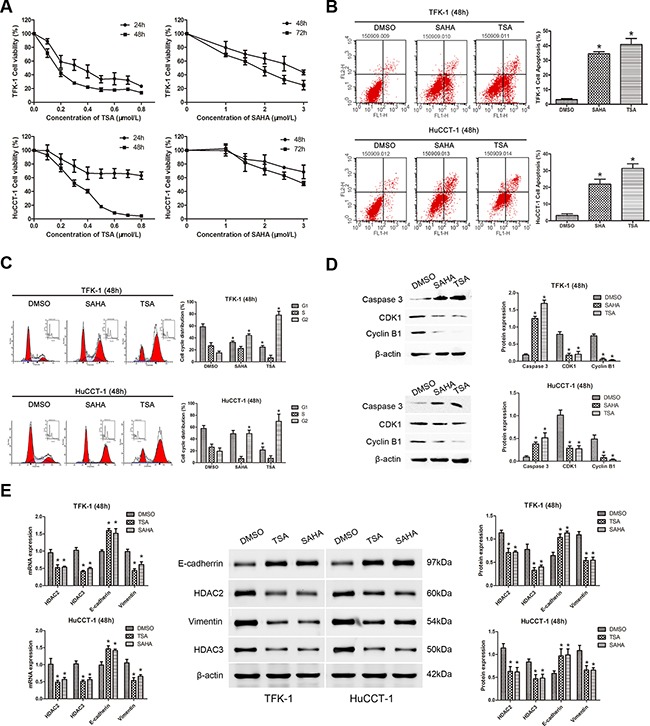
TSA and SAHA suppress cell proliferation, promote cell apoptosis, induce cell cycle arrest, and restrain EMT in CCA cell lines For all experiments, TFK-1 and HuCCT-1 cells were incubated with the indicated concentrations (IC_50_ values at 48 hours) of TSA (left panels) or SAHA (right panels). 1% DMSO treatment was used as a negative control and each experiment was repeated three times. Data are presented as means ± SD. **A.** The survival rates of TFK-1 (upper panel) and HuCCT-1 (lower panel) cells were detected by CCK-8 assay. Survival Rate % = (OD_treated_ − OD_blank_)/(OD_control_ − OD_blank_) × 100%. **B.** Apoptotic cells were analyzed by FACS via staining of annexin V. The percentage of apoptotic cells is shown (TFK-1, upper panels; HuCCT-1, lower panels; * *P*<0.05). **C.** An increased number of cells in G2/M phase was found by FACS analysis after treatment with TSA or SAHA. The percentage of TFK-1 cells in G1 phase was also markedly decreased. A decrease in the percentage of HuCCT-1 cells in G1 phase only observed after treatment with TSA (TFK-1, upper panels; HuCCT-1, lower panels; *P<0.05). **D.** Western blot demonstrated that the expression of the caspase 3 was increased, while the expression of the G2/M phase checkpoint proteins, CDK1 and cyclin B1, was down-regulated in TFK-1 (upper panels) and HuCCT-1 (lower panels) cells after treatment with TSA or SAHA. β-actin was used as the internal control. *P<0.05. **E.** qRT-PCR was used to analyze the mRNA expression of *HDAC2*, *HDAC3*, *CDH1*, and *VIM* in TFK-1 and HuCCT-1 cell lines after treatment with TSA or SAHA, *ACTB* was used as the internal control (Left panels, *P<0.05). Western blot was used to explore protein expression of HDAC2, HDAC3, E-cadherin, and vimentin in TFK-1 and HuCCT-1 cell lines after treatment with TSA or SAHA. β-actin was used as the internal control (Left panels, *P<0.05). Representative images are shown (middle panels). Statistical analysis of the relative optical density of each band is shown (right panels, *P<0.05).

To investigate the anti-cancer effects of HDACIs in CCA, TSA and SAHA were used to treat the two CCA cell lines, TFK-1 and HuCCT-1 (1% DMSO treatment was used as a negative control). Both TSA and SAHA suppressed cell proliferation in a dose-dependent manner in CCA cells (Figure 2A). At 48 hours, the IC_50_ of TSA was 0.3075 μM for TFK-1 cells and 0.4718 μM for HuCCT-1 cells, while the IC_50_ of SAHA was 3.257 μM for TFK-1 cells and 4.603 μM for HuCCT-1 cells. Moreover, when TFK-1 and HuCCT-1 cells were treated with TSA and SAHA at their respective IC_50_ doses for 48 hours, the rate of apoptosis was higher than that of the DMSO-treated cells (*P*<0.05, Figure 2B). Additionally, both drugs induced G2/M cell cycle arrest in both CCA cell lines (*P*<0.05, Figure 2C), and the percentage of TFK-1 cells in G1 phase was also markedly decreased (*P*<0.05, Figure 2C). For HuCCT-1 cells, a decrease in the percentage of cells in G1 phase was only observed after treatment with TSA (*P*<0.05, Figure 2C). Consistent with these results, expression of the well-defined apoptosis protein marker, cleaved caspase 3, was markedly increased (*P*<0.05, Figure 2D), while expression of G2/M phase checkpoint proteins, including CDK1 and cyclin B1, was down-regulated after treatment with TSA or SAHA (*P*<0.05, Figure 2D). To detect the impact of HDACIs on EMT, we also examined the expression of the epithelial marker, E-cadherin, and the mesenchymal marker, vimentin. Elevated expression of E-cadherin and reduced expression of vimentin were detected at both the mRNA and protein level after TSA and SAHA treatment. In addition, the expression of HDAC2 and HDAC3 were down-regulated (*P*<0.05, Figure 2E). These results indicated that HDAC2 and HDAC3 might restrain EMT in CCA cells.

### Identification of *TACC3* as a molecular drug target of HDAC inhibitors and its correlation with poor prognosis in CCA patients

**Figure 3:**
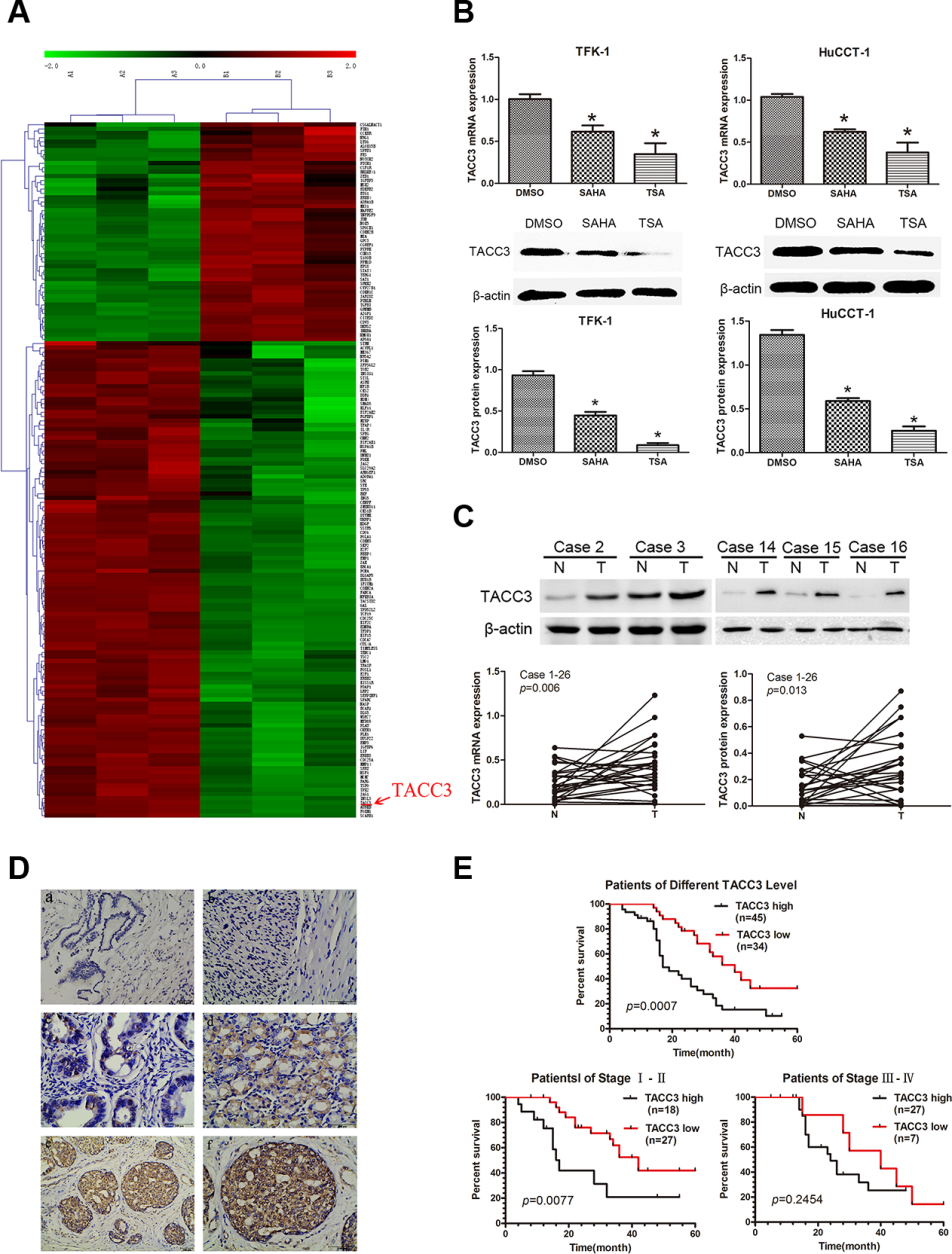
Microarray analysis indicates *TACC3* as a molecular drug target of HDAC inhibitors, and the expression of *TACC3* correlates with the prognosis of CCA patients **A.** Hierarchical clustering analysis of 163 mRNAs involved in cell proliferation and migration that were differentially expressed (Fold Change ≥ 2.0 and *P*-value ≤ 0.05) after treatment with TSA (right) compared with 1% DMSO (left), which was used as negative control. Red coloring indicates up-regulated and green indicates means down-regulated expression. **B.** Expression of *TACC3* mRNA (upper panels) and protein (lower panels) in TFK-1 and HuCTT-1 cells was validated by qRT-PCR and WB. Cells were treated with the indicated concentrations of TSA and SAHA (respective IC_50_ values at 48 hours). 1% DMSO treatment was used as negative control and β-actin was used as the internal control. These experiments were repeated three times, and data are shown as mean ± SD, *P<0.05. **C.** Expression of *TACC3* mRNA and protein in CCA samples and adjacent non-tumor bile duct tissues (n=26) was analyzed by qRT-PCR (*P*=0.006) and WB (*P*=0.013). Representative images are shown in the upper panel. β-actin (or *ACTB*) was used as the internal control, experiments were repeated three times, and data are shown as mean ± SD, *P<0.05. **D.** The expression of TACC3 in CCA tissues and adjacent non-tumor tissues (n=79) analyzed by IHC. (a) negative TACC3 staining in adjacent non-tumor bile duct tissues (200X); (b) negative TACC3 staining in CCA tissues (400X); (c) weak TACC3 staining in the cytoplasm (400X); (d) moderate TACC3 staining in the cytoplasm (400X); (e, f) strong TACC3 staining in the cytoplasm (200X,400X). Scale bar, 100 μm. **E.** Kaplan-Meier analysis showing that patients with low TACC3 expression (n=34) had longer OS than patients with high TACC3 expression (n=45; median OS: 40 months *vs* 17 months, *P*<0.001). Patients in stage I-II with low TACC3 expression (n=27) had longer OS than patients with high TACC3 expression (n=18; median OS: 42 months *vs* 17 months, *P*=0.0077). However, there was no correlation observed in patients with stage III-IV CCA (median OS: 40 months *vs* 25 months, *P*=0.2454, log-rank test).

To identify the target transcripts of HDACIs, mRNA expression profiles of TFK-1 cells treated with TSA at the IC_50_ dose for 48 hours, were measured via microarray analysis. TFK-1 cells treated with 1% DMSO were used as a negative controls. The microarray data have been stored in the NCBI GEO repository and are accessible through the following GEO accession number: GSE78867 (http://www.ncbi.nlm.nih.gov/geo/query/acc.cgi?acc=GSE78867). In total, there were 1568 up-regulated genes and 1448 down-regulated genes identified. Gene ontology (GO) and Kyoto encyclopedia of genes and genomes (KEGG) software was used to identify genes involved in cell proliferation and migration, leaving 163 genes as shown in the hierarchical clustering graph (Figure 3A). Among these genes, *TACC3* mRNA was markedly down-regulated (Fold Change=6.317668; *P*<0.0001) after TSA treatment. To validate these findings, *TACC3* mRNA expression was analyzed by qRT-PCR in CCA cell lines treated with TSA or SAHA. The qRT-PCR results confirmed that *TACC3* mRNA was down-regulated after treatment with HDACIs (*P*<0.05; Figure 3B).

Next, we investigated the expression of TACC3 protein in CCA cell lines by WB. TACC3 was also down-regulated after cells were treated with HDACIs (*P*<0.05; Figure 3B), but TSA more effectively down-regulated TACC3 expression than SAHA in CCA cells. These findings were investigated further with qRT-PCR and WB in 26 pairs of CCA tissues and non-tumor tissues. We found that TACC3 expression was higher in CCA tissues than in adjacent non-tumor tissues at both the mRNA and protein levels (*P*<0.05; Figure 3C). IHC studies on 79 paraffin-embedded CCA specimens indicated that TACC3 was localized to the cytoplasm of CCA cells (Figure 3D). The expression of TACC3 in CCA tissues was higher than in the paired adjacent non-tumor tissues (*P*=0.008; Table 1). These findings strongly suggested that *TACC3* was down-regulated after treatment with HDACIs and up-regulated in CCA tissues compared with adjacent non-tumor tissues, and that *TACC3* may be a potential anti-tumor molecular drug target of HDACIs in CCA.

To investigate whether TACC3 expression is correlated with CCA progression, we analyzed its association with the clinicopathological characteristics of CCA specimens. As shown in Table 1, there was a strong correlation between high TACC3 expression and lymph node status (*P*=0.003), TNM stage (*P*<0.001), and differentiation (*P*<0.001), but not with age (*P*=0.259) or gender (*P*=0.238). Kaplan-Meier analysis suggested that OS was shorter for patients with high TACC3 expression than for those with low TACC3 expression (*P*<0.001; Figure 3E). Furthermore, TACC3 expression was correlated with OS in patients with stage I-II disease (n=45; *P*=0.0077; Figure 3E), but not in patients with stage III-IV disease (n=34; *P*=0.2454; Figure 3E). Additionally, multivariate analysis showed that TACC3 expression was an independent prognostic factor for OS in patients with CCA (95%CI: 1.503–3.1670; *P*=0.0063; Table 2).

### Knockdown of *TACC3* suppresses the proliferation, migration, and invasiveness of CCA cells

**Figure 4:**
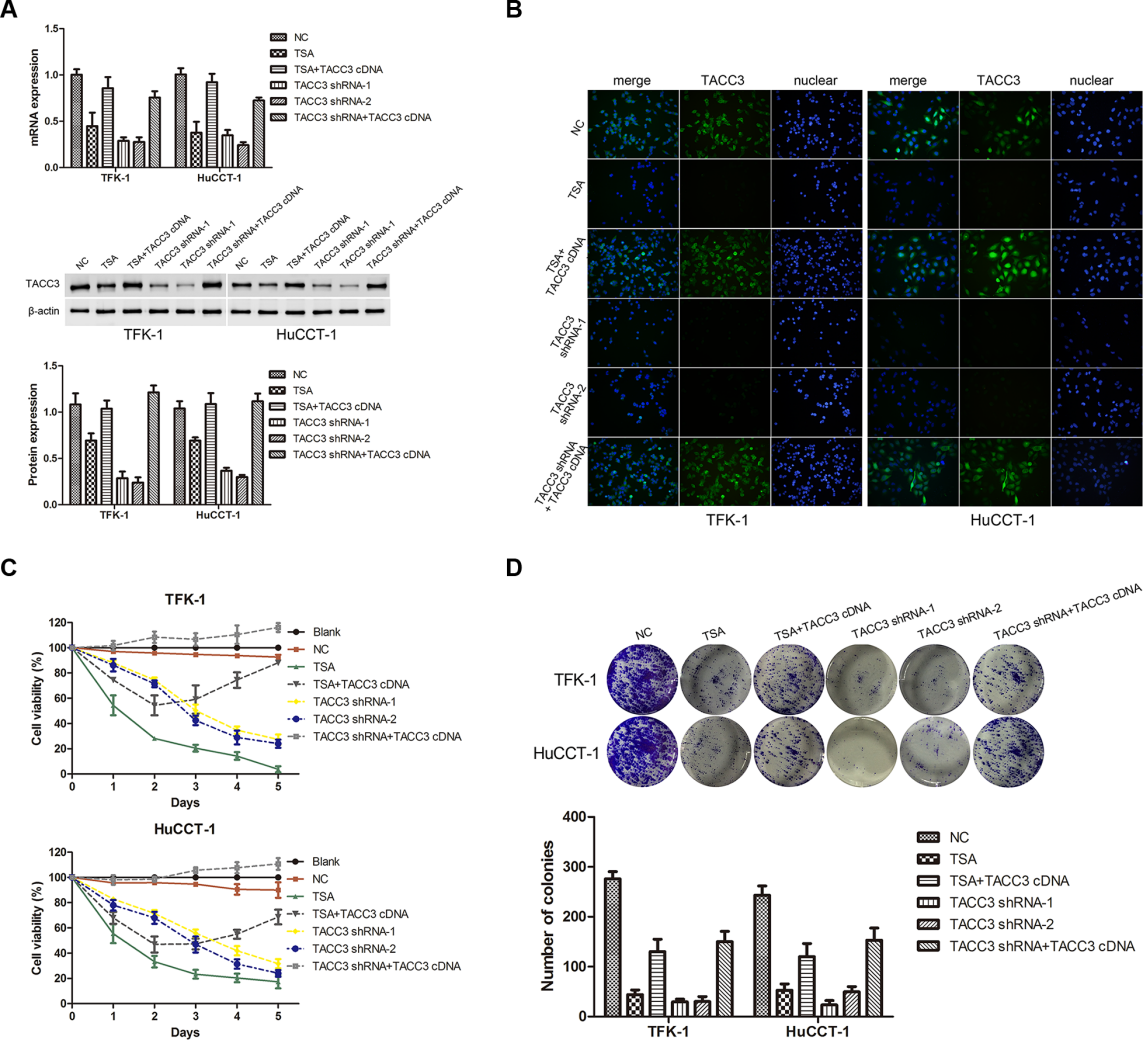
Knockdown of *TACC3* suppresses proliferation and colony formation of CCA cells TFK-1 and HuCCT-1 cells were treated with TSA or small interfering RNAs as indicated, and empty vector was used as the negative control (NC). **A.** qRT-PCR and western blot assays were used to detect the expression of TACC3. For WB analysis, representative imagines are shown (middle panels). Statistical analysis of the relative optical density of each band is shown (lower panels). β-actin was used as an internal control, experiments were repeated three times, and data are shown as mean ± SD, *P<0.05. **B.** Immunofluorescence was used to detect the expression of TACC3 (green). DAPI (blue) was used to stain the nuclei. The fluorescence intensity of TACC3 was stronger in NC groups, and was weaker in cells treated with TSA or transfected with shRNA. In the *TACC3* shRNA rescue experiment, fluorescence intensity was recovered. One representative experiment out of the three performed is shown (400X). **C.** Survival rate of TFK-1 and HuCCT-1 cells was detected by CCK-8 assay. Experiments were repeated three times and data are shown as mean ± SD. **D.** Colony formation assays were performed to evaluate the proliferative capability of TFK-1 (upper panel) and HuCCT-1(lower panel) cells. A representative image is shown, and a statistical comparison of the indicated groups was performed across three independent experiments, *P<0.05 and **P<0.001.

To investigate the potential roles of TACC3 in CCA tumorigenesis, we stably knocked down *TACC3* in TFK-1 and HuCCT-1 cells with lentiviral transfection of two *TACC3* shRNA duplexes. For the rescue experiment, lentiviruses carrying *TACC3* cDNA were re-transfected into the cells with *TACC3* shRNA or transfected into TSA-treated cells. qRT-PCR, WB, and immunofluorescence (IF) analyses confirmed that *TACC3* expression was down-regulated in cells treated with TSA or *TACC3* shRNA (*P*<0.05; Figure 4A&4B), while the expression of *TACC3* was rescued in cells transfected with *TACC3* cDNA (*P*<0.05; Figure 4A&4B). Compared with the blank and negative control (NC) groups, TSA treatment and *TACC3* shRNA treatment resulted in markedly lower cell viability (*P*<0.05; Figure 4C). TSA treatment reduced the number of colonies formed compared with the NC group (TFK-1: 44±16.5 *vs* 276±25.1 cells per well, *P*<0.001; HuCCT-1: 52.7±21.9 *vs* 243.3±32.2 cells per well, *P*<0.001), and when TSA-treated cells were transfected with *TACC3* cDNA, the number of colonies formed was increased compared with TSA treatment (TFK-1: 130.7±43.6 *vs* 44±16.5 cells per well, *P*<0.05; HuCCT-1: 120.5±27.2 *vs* 52.7±21.9 cells per well, *P*<0.05). Similarly, there was a decrease in the number of colonies formed in cells with *TACC3* shRNA knockdown compared with the NC group (TFK-1: 29.3±10.1 & 30±17.6 *vs* 276±25.1 cells per well, *P*<0.001; HuCCT-1: 23.3±15.3 & 49.7±17.9 *vs* 243.3±32.2 cells per well, *P*<0.001), while *TACC3* shRNA cells given *TACC3* cDNA had increased colony formation (TFK-1: 29.3±10.1 & 30±17.6 *vs* 150±36.1 cells per well, *P*<0.05 ; HuCCT-1: 24.3±15.3 & 49.7±17.9 *vs* 153.3±41.6 cells per well, *P*<0.05, Figure 4D). These results indicated a growth-promoting role of *TACC3* in CCA cells.

**Figure 5:**
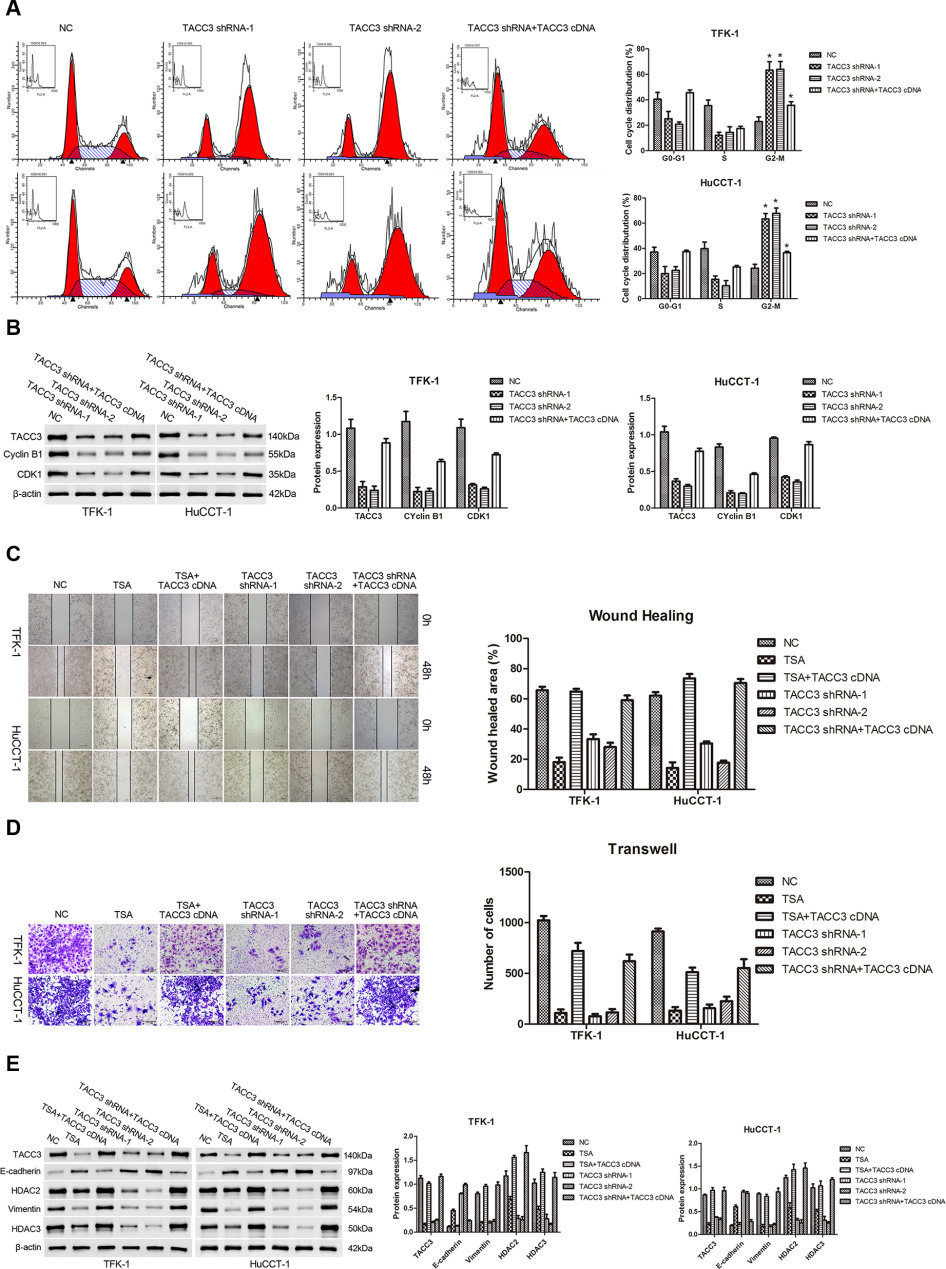
Knockdown of *TACC3* induces G2/M cell cycle arrest and suppresses the migration and invasion of CCA cells For all experiments, cells transfected with empty vector were used as negative control (NC), and experiments were repeated three times. Data are shown as mean ± SD. **A.** FACS analysis was used to investigate differences in cell cycle distribution following *TACC3* silencing or overexpression. *TACC3* silencing drove G2/M arrest in TFK-1 (upper panels) and HuCCT-1 (lower panels) cells. In addition, when *TACC3* knockdown cells were given *TACC3* cDNA, the G2/M phase distribution was decreased. *P<0.05. **B.** WB results indicated that the expression of CDK1 and cyclin B1 were down-regulated with *TACC3* knockdown, and were up-regulated when cells were given *TACC3* cDNA. Representative imagines are shown (left panels). Statistical analysis of the relative optical density of each band is shown (right panels). β-actin was used as an internal control, *P<0.05. **C.** Wound healing assays was performed to explore the migration capability, and solid lines represent the wound edges. Images were captured using light microscopy (4X). The migration index was calculated as described in the Materials and Methods (TFK-1, upper panels; HuCCT-1, lower panels). Representative images are shown (left panel). Statistical analysis is shown (right panel), *P<0.05 and **P<0.001. **D.** Transwell assay was used to investigate the invasiveness of cells. The number of cells that invaded through the membrane was determined under a light microscope (200X). Representative images are shown (left panel). Statistical analysis is shown (right panel), *P<0.05 and **P<0.001. **E.** Western blot assay was employed to investigate the expression of E-cadherin, vimentin, HDAC2, and HDAC3. Representative images are shown (left panels). Statistical analysis of the relative optical density of each band is shown (right panels). β-actin was used as an internal control, *P<0.05.

To explore the effects of TACC3 on the cell cycle, FACS was used to assess cell cycle distributions following *TACC3* silencing or overexpression. Knockdown of *TACC3* promoted G2/M arrest (*P*<0.05, Figure 5A), whereas G1/S transition was suppressed in TFK-1 and HuCCT-1 cells. In addition, when *TACC3* knockdown cells were given *TACC3* cDNA, the G2/M phase distribution was decreased (*P*<0.05, Figure 5A). Moreover, the expression of G2/M phase checkpoint proteins, CDK1 and cyclin B1 were down-regulated with *TACC3* knockdown (*P*<0.05, Figure 5B), and up-regulated when *TACC3* knockdown cells were given *TACC3* cDNA (*P*<0.05, Figure 5B). Furthermore, wound healing occurred more slowly in cells treated with TSA or transfected with *TACC3* shRNA compared with the NC cells (*P*<0.001; Figure 5C). When cells were transfected with *TACC3* cDNA, cell migration was enhanced compared with TSA treatment or *TACC3* knockdown (TFK-1: *P*<0.05; HuCCT-1: *P*<0.001; Figure 5C). Likewise, treatment with TSA or knockdown of *TACC3* expression reduced the invasiveness of CCA cells compared with the NC group (*P*<0.001; Figure 5D), while *TACC3* up-regulation enhanced cell invasion capability (*P*<0.05; Figure 5D). Finally, we used WB to assay the expression of EMT-associated proteins in TFK-1 and HuCCT-1 cells after TSA treatment or transfection with *TACC3* shRNA. Knockdown of *TACC3* increased E-cadherin expression and decreased vimentin expression (*P*<0.05; Figure 5E). Expression of HDAC2 and HDAC3, which correlated with lymphatic metastasis and prognosis in CCA patients, were also decreased (*P*<0.05; Figure 5E). With *TACC3* rescue, the expression of vimentin, HDAC2, and HDAC3 were elevated, while E-cadherin expression was decreased (*P*<0.05; Figure 5E). These results indicated that knockdown of *TACC3* suppresses the proliferation, migration, and invasion ability of CCA cells and suggested that *TACC3* is an anti-cancer molecular drug target of HDACIs.

### Targeted silencing of *TACC3* suppresses CCA tumorigenicity and metastasis *in vivo*

**Figure 6:**
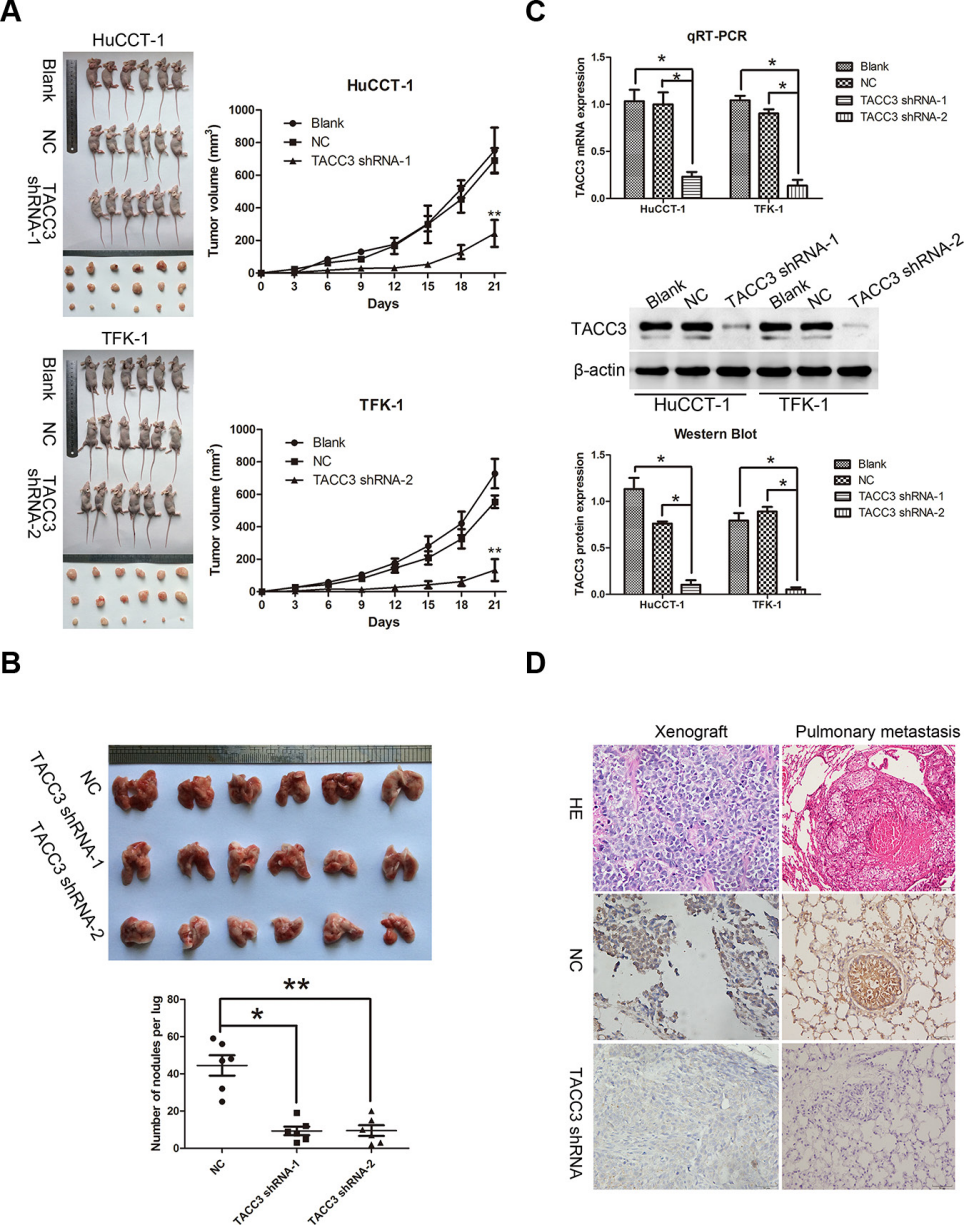
Targeted silencing of *TACC3* suppresses CCA tumorigenicity and metastasis, *in vivo.* **A.** The effects of *TACC3* silencing on tumor suppression *in vivo*. Images of tumors formed in nude mice injected subcutaneously with HuCCT-1 cells transfected with the blank, negative vector, and *TACC3* shRNA-1 (upper). Images of tumors formed in nude mice injected subcutaneously with TFK-1 cells transfected with the blank, negative vector and *TACC3* shRNA-2 (lower). Tumor growth curves are plotted (right). **P<0.001. **B.** A pulmonary metastasis model was established after 6 weeks of the indicated treatment. Images from the pulmonary metastasis model (upper panel) and the corresponding statistical analysis (lower panel) are shown. *P<0.05 and **P<0.001. **C.** qRT-PCR (upper panel) and WB (middle and lower panel) were used to assess *TACC3* mRNA and protein expression in tumor xenografts. *P<0.05. **D.** IHC was used to detect the expression of TACC3 in tumor xenografts and pulmonary metastasis tumor tissues (400X). Scale bar, 100 μm.

To further investigate the potential therapeutic role of TACC3 in CCA, a subcutaneous tumor model was established in nude mice. Xenograft tumors grown from blank and NC cells had larger mean volumes and weights and were frequently more aggressive than tumors grown from cells transfected with *TACC3* shRNA (HuCCT-1, *P*<0.001; TFK-1, *P*<0.001; Figure 6A). Next, a pulmonary metastasis tumor model was generated by injection of tumor cells into the tail vein of nude mice. Fewer metastatic nodes were detected in the pulmonary tissues of the treatment groups (TFK-1: *TACC3* shRNA-1, *P*<0.05; *TACC3* shRNA-2, *P*<0.001; Figure 6B). qRT-PCR, WB and IHC were performed to verify knockdown of *TACC3*. Expression of *TACC3* was decreased in subcutaneous tumors formed by cells that were transfected with *TACC3* shRNA (*P*<0.05; Figure 6C&6D). These results indicated that targeted *TACC3* knockdown suppresses tumor growth and metastasis of CCA cells, *in vivo*.

## DISCUSSION

CCA is the second most common hepatobiliary malignancy [1] and is one of the most life-threatening diseases due to its aggressiveness and metastatic tendencies [36]. In spite of substantial progress, we still lack a valid biomarker that is involved in tumor proliferation, invasion, and metastasis in patients with CCA. Therefore, it is urgent to find a new molecular target for therapy and to predict the prognosis of patients with CCA. Many studies have reported the differential expression of specific HDAC isoforms in a variety of hematological or solid malignancies and cell lines, such as lymphoma [14], breast [15], gastric [16], colorectal [17], and lung cancers [18], hepatocellular carcinoma [19], and cholangiocarcinoma [20]. Class I and II HDACs are overexpressed in a variety of CCA cell lines; however, few studies have investigated HDAC expression status and its prognostic value in CCA patients [20]. In this study, we investigated the expression of HDACs 1-10 and their biological significance in CCA. We found that HDACs 2, 3 and 8 were up-regulated at the mRNA and protein levels in CCA tissues compared with adjacent non-tumor tissues. In addition, the expression of HDACs 2 and 3 proteins, but not HDAC8, was positively correlated with lymph node metastasis, TNM stage, and differentiation. Moreover, patients with high expression of HDAC2 or HDAC3 (or both) had shorter OS. These results indicated that HDAC2 and 3 may serve as useful prognostic biomarkers and that the modulation of the histone acetylation may be a novel therapeutic strategy for CCA. HDACIs have the potential to disrupt multiple signaling pathways to inhibit tumor growth and induce apoptosis [37, 38]. Our data showed that TSA and SAHA inhibited the proliferation of CCA cells and induced apoptosis as well as G2/M cell cycle arrest. Mottamal et al. reported that Epoxide 1-Alaninechlamydocin isolated from *Tolypocladium sp.* also elicited potent anti-proliferative effects, induction of G2/M cell cycle arrest, and apoptosis in human cancer cells [39]. Our results are consistent with those of an earlier report that claimed that TSA induced a delay in G2/M transition of HeLa cells via the downregulation of Cyclin B1, PIK1 and Survivin, and via the upregulation of p21^WAF1/CIP1^ [40].

Furthermore, to identify the target transcripts of HDACIs that may be potential regulators of the suppression of CCA tumorigenesis, we determined mRNA expression profiles by microarray analysis. The microarray results revealed that the expression of *TACC3* was down-regulated in CCA cell lines upon treatment with TSA, a finding confirmed by qRT-PCT and WB. Until recently, it has been unclear whether *TACC3* acts as an oncogene or a tumor suppressor gene. Accumulating evidence indicates that alterations in *TACC3* expression depend on the organ and type of cancer [30–35]. In this study, we found that *TACC3* was overexpressed in CCA tissues and was positively correlated with lymph node metastasis, TNM stage, and differentiation. In addition, patients whose tumors had high TACC3 expression exhibited a shortened survival time, and this correlation was more obvious in patients with stage I-II cancers compared with patients with stage III-IV cancers. Our study demonstrated that TACC3 may be a potential anti-cancer molecular drug target of HDACIs and a potential prognostic indicator for CCA.

Recently, various reports have suggested that TACC3 may be a potential therapeutic target and that the targeted knockdown of *TACC3* can inhibit tumor cell proliferation and induce apoptosis. *TACC3* overexpression is associated with defective checkpoint control and impaired DNA repair systems, resulting in genomic instability [41]. Additionally, TACC3 participates in the regulation of cell proliferation, differentiation, and transcription [27]. HDACIs decrease ionic interactions between DNA and histones, which results in chromatin relaxation and the subsequent acceleration of DNA transcription. However, the exact relationship of HDACIs and *TACC3* has not yet been elucidated. In this study, we demonstrated that HDACIs downregulate *TACC3* expression and that targeted *TACC3* knockdown suppresses CCA cell proliferation and colony formation, and induces G2/M phase arrest *in vitro*. These findings are in agreement with previous studies that down-regulated *TACC3* inhibits cell growth in esophageal cancer [42]. In addition, *TACC3*-depleted cells were highly sensitive to paclitaxel-induced cell death, which occurred even when the levels of active Akt and p21 were high [43]. Moreover, Yim et al. also reported that *TACC3* knockdown combined with paclitaxel treatment led to a synergistic acceleration of G2/M phase arrest and apoptosis in HeLa cells [44]. Taken together, these findings indicate that TACC3 may be a potential anti-cancer molecular target either in combination with other drugs or alone.

EMT is a pivotal process in the early phase of the metastasis cascade and can be initiated by various signaling pathways [45]. Up-regulated expression of *TACC3* promotes EGF-mediated EMT via the initiation of the PI3K/Akt and ERK signaling transduction pathways [27, 29]. To investigate whether alterations in *TACC3* gene expression impact the ability of CCA cells to invade and migrate, wound-healing and Transwell assays were performed. We found that knockdown of *TACC3* reduced the invasive and migratory ability of CCA cells, while increased *TACC3* expression increased invasion and migration. In addition, elevated expression of the epithelial marker, E-cadherin, and reduced expression of the mesenchymal marker, vimentin, after *TACC3* knockdown verified the role of TACC3 in EMT. These findings indicated that TACC3 might act as an important regulator of CCA metastasis.

Based on our interesting *in vitro* findings, we next established subcutaneous and pulmonary metastasis tumor models to further investigate the potential therapeutic role of TACC3 in CCA, *in vivo*. We found that targeted silencing of *TACC3* markedly inhibited xenograft tumor growth and the formation of pulmonary metastatic nodes, suggesting that down-regulation of *TACC3* suppresses EMT-induced invasion and metastasis. Another study demonstrated that *TACC3* suppression causes tumor regression and leads to embryonic lethality in mice due to massive apoptosis in tumor, but not normal, tissues [46]. Therefore, the targeted silencing of *TACC3* may be a valid approach for anti-tumor therapy for CCA; however, its mechanism still needs further experimental exploration.

In summary, we observed that HDACs 2, 3, and 8 were over-expressed in CCA tissues and that HDACs 2 and 3 may serve as useful prognostic biomarkers for CCA. The HDACIs, TSA and SAHA, inhibited the proliferation of CCA cells and induced apoptosis and G2/M cell cycle arrest. Microarray analysis demonstrated that TSA led to the down-regulation of *TACC3*, and that patients with CCA with a high TACC3 expression were predicted to have a poor prognosis. Therefore, targeted *TACC3* silencing may be a valid approach for anti-tumor therapy for patients with CCA.

## MATERIALS AND METHODS

### Patients and tissue specimens

Two independent series including 105 patients with CCA were enrolled in this study. Group 1: 4% paraformaldehyde-fixed, paraffin-embedded paired tissues, which included CCA samples and adjacent non-tumor tissues (2 cm away from the tumor boundaries) were obtained from 79 CCA patients who underwent resection between January 2008 and July 2010 at the Department of Biliary and Pancreatic Surgery, Tongji Hospital, Tongji Medical College, Huazhong University of Science and Technology, Wuhan, China. The clinicopathological information of the 79 patients with CCA is listed in Supplementary Table S1. Group 2: twenty-six fresh CCA samples and adjacent non-tumor tissues were collected from the same hospital between January 2014 and December 2014. None of the patients had received any chemotherapy or radiotherapy prior to surgery. All diagnoses were confirmed by 2 pathologists at Tongji Hospital. Tumor staging was based on the TNM classification standard of the 7^th^ edition of the UICC/AJCC staging manual. All patients signed an informed consent before surgery, and the ethics approval for this study was obtained from the Tongji Hospital Research Ethics Committee (Supplementary Figure S1).

### Cell lines

Two human CCA cell lines, TFK-1 and HuCCT-1, were kindly provided by Professor Peter Schemmer (Department of General and Transplant Surgery, University Hospital Heidelberg, Germany). The cells were cultured in RPMI-1640 (Gibco, CA, USA) supplemented with 10% fetal calf serum (Gibco, CA, USA), 100 U/ml penicillin and 100 μg/ml streptomycin (Beijing Solarbio Science & Technology Co., Beijing, China) and were maintained at 37°C in an incubator with 5% CO_2_.

### Quantitative real-time polymerase chain reaction (qRT-PCR) analysis

Total RNA was isolated from tissue specimens or from cell lines using TRIzol reagent (Life Technologies, CA, USA) according to the manufacturer's instructions. Complementary DNA (cDNA) was synthesized using 2 μg of the total RNA according to the instructions of the reverse transcriptase kit (Takara Bio, Inc., Dalian, China) in a LifePro Thermal Cycler (Hangzhou Bioer Technology Co. Ltd., Hangzhou, China). Then, cDNA samples (2 μl) were subjected to qRT-PCR using a SYBR^®^ Premix EX Taq kit (Takara Bio, Inc., Dalian, China) for 40 cycles in a CFX Connect^TM^ Real-Time System (Bio-Rad, Hercules, CA, USA). *ACTB* was used as an internal control. Primers were designed and synthesized by Shanghai Sango Biotech Co. Ltd., Shanghai, China (listed in Supplementary Table S2). The cycle threshold (Ct) of different genes was first normalized to *ACTB* for the same sample, and fold changes were calculated through relative quantification (2^−ΔΔCt^).

### Western blotting (WB) analysis

WB analysis was performed as previous described [47, 48]. As an internal control, blots were incubated with antibodies to glyceraldehyde-phosphate dehydrogenase (GAPDH) or β-actin. The primary antibodies used are listed in Supplementary Table S3. To quantify the relative levels of protein expression, the intensity of the specific bands was estimated using the Image J2X analysis software package (National Institute of Mental Health, Bethesda, MD, USA).

### Immunohistochemistry (IHC)

The streptavidin biotin compound (SABC) method was used to detect the expression of HDAC2, 3, 8 and TACC3 proteins, as previously described [47]. The sections were incubated with a polyclonal or a monoclonal antibody overnight at 4°C in a moist chamber at the indicated dilutions (listed in Supplementary Table S3). The next day, the sections were incubated with the appropriate secondary antibody for 30 min at 37°C. Finally, the sections were incubated with streptavidin-biotin complex (Boster Biotech, Wuhan, China) at a dilution of 1:100 for 5 min and counterstained with Mayer's hematoxylin, which stains the nucleus. The slides were dehydrated, mounted, and observed by microscopy. IHC scores were assessed by 3 pathologists who were blinded to the patient conditions, as previously described [47].

### Microarray analysis

TFK-1 cells that were treated with 1% DMSO and TSA (0.3 μmol/L) for 48 hours were selected for microarray analysis; cells selection occurred in triplicate. Total RNA was isolated from cell lines using TRIzol reagent (Life Technologies, CA, USA), the concentration of RNA in the samples was detected by a NanoDrop ND-2000 (Thermo Scientific) and RNA integrity was assessed by an Agilent Bioanalyzer 2100 (Agilent Technologies). Briefly, cDNA was synthesized and labeled (Cyanine-3-CTP) before it was purified and hybridized to the microarray (Agilent SurePrint G3 Human Gene Expression Array, 8*60K, Design ID: 039494). After washing, the arrays were scanned by an Agilent Scanner G2505C (Agilent Technologies). Original data were extracted using Feature Extraction software (version 10.7.1.1, Agilent Technologies). Further data analysis was conducted using Agilent Genespring software (version 12.5). To identify significant differential expression of mRNAs, we performed a Volcano Plot filtering (Fold Change >=2.0 and *P*-value <=0.05) between the DMSO group and the TSA-treated group. After that, the significantly differentially expressed genes were further analyzed by GO and KEGG software to judge their biological function and the pathways through which they function. Finally, hierarchical clustering was performed based on the significant differential expression of mRNAs, and a thermograph was used to demonstrate the expression patterns of the differentially expressed genes between both groups.

### Cell viability assay *in vitro*

A CCK-8 kit (Dojindo Laboratories Co. Ltd, Kumamoto, Japan) was used as a colorimetric assay to assess cell viability. Briefly, cells (5 × 10^3^ cells/well) were seeded into 96-well plates with 100 μl per well of RPMI-1640 culture medium supplemented with 10% FBS and the indicated reagents. Each sample had six replicates. At the indicated time points, the medium was replaced by 100 μl fresh culture medium, and 10 μl CCK-8 solution was added to each well. Plates were incubated for 1-4 hours at 37°C before the absorbance was recorded at 450 nm using a Quant ELISA Reader (BioTek Instruments, USA). The percentage of viable cells was calculated according to the following method in a previously published report [48]: Survival Rate % =(OD_treated_ − OD_blank_)/(OD_control_ − OD_blank_) × 100%. The IC_50_ was calculated using probit regression analysis. The tests were repeated at least 3 times.

### Flow cytometry

Cells (5 × 10^3^ cells/well) were seeded into 6-well plates and allowed to adhere overnight. Then, the medium was replaced by 2 ml fresh experimental medium containing TSA or SAHA at different concentrations according to the 48-hour IC_50_ values. After 48 hours, cells were harvested, and the rate of apoptosis was determined by an Annexin V-FITC/PI apoptosis detection kit (KeyGen Biotechnology Co., Ltd., Nanjing, China) according to the manufacturer's instructions. Data analysis was performed using FlowJo software. For cell cycle analysis, after 48 hours the cells were fixed in 70% ethanol overnight at 20°C, stained with propidium iodide, and then analyzed by a FACScan flow cytometer (Biosciences, San Jose, CA, USA) and ModFit 3.0 software (Verity Software House, Topsham, ME, USA).

### Lentiviral vector construction and establishment stable cell clones

Three recombinant lentiviruses containing *TACC3* (GenBank access number: NM_006342.2) expressing *TACC3*-shRNA-1 (GCATGCACGGTGCAAATGA), *TACC3*-shRNA-2 (CCACAGATCTGAACTCCAT) and *TACC3*-specific cDNA were purchased from Genechem Co., Ltd (Shanghai, China). The GV248 vector (hU6-MCS-Ubiquitin-EGFP-IRES-puromycin) used for the stable expression of shRNA against *TACC3* and a fluorescent marker (GFP-RFP fusion protein) contained a puromycin resistance gene. The negative control (NC) sequence was indicated as “NC” and had no homology to any human genomic sequences. In addition, the cDNA of the human *TACC3* gene, a fragment encoding the *TACC3*-sequence plus 1439 bp at both 5’- and 3’- flanking regions was amplified with the primers 5’- GAGGATCCCCGGGTACCGGTCGCCACCATGAGTCTGCAGGTCTTAAACGAC-3’ (forward) and 5’- TCCTTGTAGTCCATACCGATCTTCTCCATCTTGGAGATGAG-3’(reverse) by PCR from human genomic DNA and then cloned into the AgeI/NheI sites of GV358. Lentiviral transfection was conducted according to the GenePharma Recombinant Lentivirus Operation Manual (http://www.genepharma.com). TFK-1 and HuCCT-1 cells (1 × 10^5^ cells/well) were seeded into 6-well plates for 24 hours, and after the addition of polybrene (8 μg/ml), the cells were infected with 2 μl of concentrated lentivirus for 72 hours. Cells were selected for 2 weeks with the addition of puromycin (5 μg/ml, Sigma-Aldrich, St, Louis, USA) to generate stable monoclonal cell lines. For the rescue experiment, stable cells with *TACC3* shRNA or cells treated with TSA (0.3 μmol/L) for 48 hours were grown in 6-well plates, and re-infected with 2 μl of concentrated lentivirus for 72 hours. Cells were selected for 2 weeks by the addition of puromycin to generate stable monoclonal cell lines. The expression of *TACC3* was confirmed by qRT-PCR, WB, and immunofluorescence.

### Immunofluorescence (IF) assay

Cells grown on cover slips in 6-well plates were fixed in 4% paraformaldehyde for 15 minutes at room temperature and then permeabilized by treatment with 0.4% Triton X-100 (Amresco, OH, USA) for 10 minutes. After blocking with 1% BSA (Amresco, OH, USA) in 1X PBS (2 ml) for 30 minutes at 37°C, the cells were incubated at 4°C overnight with the primary antibody (TACC3, 1:50). Then, the cells were rinsed and incubated for 1 hour at 37°C with fluorescein (FITC)-conjugated Affinipure goat anti-rabbit IgG (H+L; 1:20, ProteinTech Group) as the secondary antibody. After the cells were washed in PBS, the nuclei were stained by DAPI (5 μg/ml, Beyotime Institute of Biotechnology) for 2 minutes at room temperature. Images were captured using a fluorescence microscope (OLYMPUS, Japan).

### Colony formation assay

Cells (500 cells/well) were seeded into 6-well plates and cultured for 2 weeks. After fixation in 4% paraformaldehyde for 10 minutes, cells were stained with 1% crystal violet. Colonies with diameters greater than 100 μm were counted, and experiments were run independently in triplicate.

### Wound healing assay

Cells were seeded into 6-well plates and cultured until they reached sufficient confluence. The cell monolayers were scratched manually with a 200 μl pipette tip. The plates were washed with PBS twice to remove floating cells. Cells were then incubated in RPMI-1640 supplemented with 1% FBS for 48 hours after the scratches were generated. Images of 6 random fields were captured by phase contrast microscopy (Nikon Corporation) for quantitative analysis. The area into which the cells migrated was measured using the Image Pro Plus v6.0 software package (Media Cybernetics Inc., Bethesda, MD, USA).

### Transwell assay

Transwell chambers (Corning NY, USA) were pretreated with a mixture of RPMI-1640 and BD Matrigel (BD Biosciences, NJ, USA) (90 μl; 8:1), which served as the basement membrane. After the membrane was hydrated in 0.1% BSA, cells (1 × 10^5^ cells/chamber) were seeded into the top chamber with 200 μl RPMI-1640 supplemented with 0.2% FBS. After 48 hours, cells were fixed and stained. Cell counts were performed using the Image-Pro Plus v6.0 software package (Media Cybernetics Inc., Bethesda, MD, USA). Each group of cells was counted in triplicate.

### *In vivo* tumorigenicity and metastasis assays

For the tumorigenicity assay, TFK-1 and HuCCT-1 cells (2 × 10^6^ cells, suspended in 100 μl RPMI-1640 without FBS) were subcutaneously injected into the upper right flank of nude mice (4-6 week-old, BALB/c/nu, female). Tumor sizes were measured by a vernier caliper every three days. Mice were sacrificed three weeks after the injection of the cells. For the metastasis assay, TFK-1 cells were injected into the tail vein of nude mice. After 6 weeks, mice were sacrificed, and the metastatic nodes in the lungs were examined by necropsy and counted. All of the *in vivo* experiments were performed in specific pathogen-free (SPF) conditions with the approval of the Committee on the Ethics of Animal Experiments of Tongji Medical College. Details are provided in the Supplementary Figure S2.

### Statistical analyses

Quantitatative data are presented as the means ± the standard deviation (SD). Significance was assessed using 2-tailed Student's t-test, analysis of variance (ANOVA), or Pearson's correlation test, when applicable. Categorical data were analyzed by the χ^2^ test. Kaplan-Meier and log-rank analyses were used to assess survival among the subgroups. A Cox proportional hazards model was used to determine the independent survival factors based on the variables selected in the univariate and multivariate analyses. P values <0.05 were considered statistically significant. All analyses were performed with SPSS 19.0 by the Statistics Teaching Room of Tongji Medical Collage, HUST.

## SUPPLEMENTARY FIGURES AND TABLES


