# Supplementary material for: TACC3 overexpression in cholangiocarcinoma correlates with poor prognosis and is a potential anti-cancer molecular drug target for HDAC inhibitors
Source: Oncotarget. 2016 Sep 26;7(46):75441–56. doi: 10.18632/oncotarget.12254 (PMC5342751; doi:10.18632/oncotarget.12254)
Supplement: Supplementary file 1 [file oncotarget-07-75441-s001.pdf]

TACC3 overexpression in cholangiocarcinoma correlates with poor prognosis and is a potential anti-cancer molecular drug target for HDAC inhibitors

SUPPLEMENTARY FIGURES AND TABLES

华中科技大学同济医学院附属同济医院伦理委员会批准件

Tongji Hospital, Tongji Medical College，Huazhong University of Science and Technology

Institutional Review Board Approval

批准号 IRB ID：TJ-C20131215

项目名称<br>Project Name	筛选胆管癌 EMT 过程中抗肿瘤效应的核心分子靶点<br>Screening the core molecular targets of anti-tumor effect in the process of cholangiocarcinoma's EMT		
申请科室<br>Applicant Department	胆胰外科<br>Department of Biliary and Pancreatic Surgery		
项目负责人<br>Principal Investigator	王剑明<br>Jianming Wang	职称<br>Title	教授<br>Professor
报送材料<br>Submitted Materials	课题研究方案<br>Research Protocol	有/Yes■ 无/No□	
	观察记录表<br>Observation Records	有/Yes■ 无/No□	
	研究人员名单<br>Researchers List	有/Yes■ 无/No□	
审查<br>Review Items	研究者资格<br>Researchers Qualifications	符合要求/Qualified ■<br>不符合要求/Not Qualified □	
	课题研究方案<br>Research Protocol	适合/Appropriate ■<br>不适合/Not Appropriate □	
有效期<br>Valid Date	2014 年 1 月至 2015 年 12 月<br>From 01/2014 to 12/2015		

评审意见/ Review Approval：

王剑明的上述研究涉及到受试者的病情隐私。经华中科技大学同济医学院附属同济医院医学伦理委员会审议研究方案及知情同意书，一致认为该研究方案设计符合《赫尔辛基宣言》原则，充分尊重受试者及其家属的知情同意权。研究过程中使用的实验手段先进、科学，采样过程符合医疗常规，并制定了受试者保护方案。

The Jianming Wang' study concerning the client's right to privacy. The study was approved by the Ethical Committee of Tongji Hospital, Tongji Medical College, Huazhong University of Science and Technology and was conducted according to the principles of the Declaration of Helsinki. Written informed consent was obtained from the subject, and his study considered Declaration of Helsinki as a statement of ethical principles.

华中科技大学同济医学院附属同济医院伦理委员会

Ethical Committee of Tongji Hospital, Tongji Medical College, Huazhong University of Science and Technology

日期/Date: 2013.12.26

Supplementary Figure S1: Ethical Approval of Clinical Research.

华中科技大学同济医学院附属同济医院实验动物伦理批准件

Tongji Hospital, Tongji Medical College ,Huazhong University of Science and Technology
Institutional Review Board Approval of Experimental Animals
批准号 IRB ID：TJ-A20131214

项目名称 Project Name	筛选胆管癌 EMT 过程中抗肿瘤效应的核心分子靶点 Screening the core molecular targets of anti-tumor effect in the process of cholangiocarcinoma's EMT		
申请科室 Applicant Department	胆胰外科 Department of Biliary and Pancreatic Surgery		
项目负责人 Principal Investigator	王剑明 Jianming Wang	职称 Title	教授 Professor
报送材料 Submitted Materials	课题研究方案 Research Protocol	有/Yes■ 无/No□	
	观察记录表 Observation Records	有/Yes■ 无/No□	
	研究人员名单 Researchers List	有/Yes■ 无/No□	
审查 Review Items	研究者资格 Researchers Qualifications	符合要求/Qualified ■<br>不符合要求/Not Qualified □	
	课题研究方案 Research Protocol	适合/Appropriate ■<br>不适合/Not Appropriate □	
有效期 Valid Date	2014 年 1 月至 2015 年 12 月 From 01/2014 to 12/2015		

评审意见/ Review Approval：

本伦理委员会审阅并讨论了上述相关材料，该课题研究符合《湖北省实验动物管理条例》和《华中科技大学实验动物伦理委员会章程》。经伦理委员会审核，同意该课题实施。

The Ethical Committee reviewed and discussed the materials related .The study was conducted according to 《Experimental animals administrative regulations of Hubei Province》and《Guidelines for Experimental Animal Ethical Committee of Huazhong University of Science and Technology》. The study is approved by The Ethical Committee.

华中科技大学同济医学院附属同济医院伦理委员会
Institutional Ethical Committee of Tongji Hospital, Tongji Medical College,
Huazhong University of Science and Technology
日期/Date：27/12/2013

Supplementary Figure S2: Ethical Approval of Experimental Animals.

Supplementary Table S1: Clinicopathological characteristics of 79 CCA patients (Group 1)

Characteristics	n = 79	
	number	percentage
**Age (y)**		
≤ 60	45	56.96%
> 60	34	43.04%
**Gender**		
male	45	56.96%
female	34	43.04%
**Differentiation**		
well	27	34.18%
moderately/poorly	52	65.82%
**Nodal invasion**		
Negative	48	60.75%
Positive	31	39.25%
**TNM stage**		
I - II	45	56.96%
III - IV	34	43.04%

Supplementary Table S2: Primer sequence used in this study

Gene	Forward(5'-3')	Reverse(5'-3')
HDAC1	GGCTGGCAACGGCAACTAT	CACTGTAAGACCACCGCACT
HDAC2	TTACTGATGCTTGGAGGAGGT	TGGCAACTCATTGGGAATCT
HDAC3	TCCACTACGGAGCTGGACACCC	TCCTCGGAGTGGAAGCGGCA
HDAC4	ACCACATGCCCAGCACGGTGGA	AGTGAGAACTGGTGGTCCAGGCG
HDAC5	AGGGCTCCACAGAGAGTGAG	CTCGTCCTTAACCTGGATGC
HDAC6	GGCTTCAGTTTCCTGTGCTC	CTATGTCCTCCTCCATGTTG
HDAC7	GAGCTTCATTCCTCCCCTGC	ACCACAGCGAAACCATTCTT
HDAC8	AAGTCCCATCCATTCCTTCA	CCAGTTCCTGCTCCTCTGAT
HDAC9	GCCCATCTCACCTTTAGACC	TTCTGGATTTGTTGCTGCTG
HDAC10	GGAGGAGTCTGTGGCTGATC	GATGCTGCTCAGGAAACCAC
TACC3	CCAGTACAGCCAGAAGGACC	CATGGCCTGGTACACAACCT
E-cadherin	CTGGACAGGGAGGATTTTGA	ACCTGAGGCTTTGGATTCCT
Vimentin	AGAGAACTTTGCCGTTGAAGC	ACGAAGGTGACGAGCCATT
β-actin	CTCTTCCAGCCTTCCTTCCT	ATGCTATCACCTCCCCTGTG

Supplementary Table S3: Information of antibodies used in this study

Antibody	WB	IHC	IF	Specificity	Company
HDAC2	1: 1000	1: 250	/	Rabbit monoclonal	Abcam
HDAC3	1: 1000	1: 250	/	Rabbit monoclonal	Abcam
HDAC8	1: 2000	1: 50	/	Rabbit monoclonal/ polyclona	Abcam
TACC3	1: 1000	1: 100	1: 50	Rabbit monoclonal	Abcam
Caspase3	1: 1000	/	/	Rabbit polyclonal	Santa Cruz
CDK1	1: 1000	/	/	Rabbit monoclonal	Abcam
Cyclin B1	1: 1000	/	/	Rabbit monoclonal	Abcam
E-cadherin	1: 500	/	/	Rabbit polyclonal	ProteinTech Group
Vimentin	1: 500	/	/	Rabbit polyclonal	ProteinTech Group
GAPDH	1: 2000	/	/	Rabbit polyclonal	Abcam
β-actin	1: 500	/	/	Rabbit polyclonal	Cell Signaling Technology
